# How Tubercles Are Formed

**Published:** 1878-05

**Authors:** 


But to return to the new-born *tubercle.* How does it destroy the lungs? In going into an apple orchard, some trees appear to be well filled with fruit, equally distributed. Other trees have bunches of apples in patches, and by reason of varied exposure to the sun, we observe apples ripened in one spot, ripening in another, and quite green in a third. So it is, if we could see the lungs of people. In some, tubercles are thickly and equally distributed over the lungs. In others they are scattered about, a patch here, another there, a third yonder. A patch ripening in the first place; just beginning to turn in the second; while in the third, they are young and hard, and may never be different. A blackberry patch is a good and useful illustration of this point. It is the key which unlocks all the mysteries of quackery. There is a truth here, which every consumptive should understand, for there is more curative virtue in it, than in all medicine. It is wonderful how it has been lost sight of professionally. It is amazing how people won't see it. And the honest physician remains but a Cassandra still—a prophet, whose teachings, truth as they are, are wholly disregarded. But more of this in another place.

As an apple grows, it takes up more room, and soon touches its neighbor. Tubercles increase, meet, soften, and rot away together, eating up the lungs as they go. That is consumption. But what makes them grow, and what makes them soften and decay away together? The nascent crude tubercle may remain stationary for half a century; may be inappreciably hurtful; may and does remain innocuous for a life-time; may be as harmless to the system as powder is harmless, if fire is kept away. In proof of this a fact is stated—a fact of every-day occurrence in the dissecting-room. Out of fifty people, dead of other diseases than consumption, and being over forty years of age, scarcely one will be found who has not more or less tubercles in the lungs. This important fact is conclusive as to one interesting point: tubercles do not necessarily destroy life, as they may lay dormant for a life-time.

But what causes the tubercles to enlarge, soften, and rot the lungs away? Instead of writing down a long list of specifications, some of which might be omitted and many forgotten, it is of prime importance to notice one effect, instead of a hundred different causes.

